# The centre cannot hold

**DOI:** 10.1107/S2053229622009020

**Published:** 2022-09-13

**Authors:** Arthur Mar

**Affiliations:** a Department of Chemistry, University of Alberta, Edmonton, Alberta T6G 2G2, Canada

**Keywords:** noncentrosymmetric, diamond-like semiconductors, nonlinear optical materials

## Abstract

Craig *et al.* [*Acta Cryst.* (2022), C**78**, 470–480] report on the synthesis of the quaternary sulfide Li_4_CdGe_2_S_7_, which is remarkable in several respects.

An enduring paradigm in solid-state chemistry is that the structures of many ionic solids can be built up by placing cations within the inter­stitial sites of a stacked arrangement of close-packed anions. Filling half the tetra­hedral sites results in structures containing corner-shared tetra­hedra as found in diamond: a zincblende (or sphalerite) family based on cubic closest packing and a wurtzite family based on hexa­gonal closest packing (Parthé, 1965 ▸). The names, taken from the polymorphs of ZnS, draw attention to the fact that most of these compounds are sulfides or selenides. When several types of metal cations are introduced, the possibilities for how they can be distributed become immense, depending on the occurrence of order or disorder. Determining the correct site distribution is essential for understanding the relation of structure to physical properties. These compounds are typically semiconducting and have attracted enormous attention over the years as attractive candidates for materials applications. Among these ‘diamond-like semiconductors,’ Cu(In,Ga)S_2_ is already well established in thin-film solar cell technology (Ramanujam & Singh, 2017 ▸) and Cu_2_ZnSnS_4_ is a promising lead-free alternative to halide perovskite solar cells (Wallace *et al.*, 2017 ▸). New classes of thermoelectric materials based on similar compounds are being investigated (Miller *et al.*, 2018 ▸). In a related application that takes advantage of the ability to control band gaps, nonlinear optical (NLO) materials based on these diamond-like semiconductors can convert coherent light to other desired wavelengths, as in the operation of a laser (Liang *et al.*, 2017 ▸). Existing NLO materials made of oxides that function in the UV–visible region are well developed, but NLO materials made of sulfides or selenides would have smaller band gaps, making them suitable for the IR region. An IR NLO laser could then be used for medical treatment or nighttime illumination. As an essential criterion for a compound to exhibit NLO behaviour, its structure should be noncentrosymmetric.

As part of their longstanding investigations of diamond-like semiconductors as IR NLO materials, Craig *et al.* (2022 ▸) report the synthesis of the quaternary sulfide Li_4_CdGe_2_S_7_, which is remarkable in several respects. First, it demonstrates the compositional richness that has yet to be fully realized among diamond-like semiconductors. Most quaternary representatives have the composition I_2_–II–IV–VI_4_ (as in the popular kesterites Cu_2_ZnSnS_4_ mentioned above), whereas those with the composition I_4_–II–IV_2_–VI_7_ are rare and further examples surely remain to be discovered. Second, the authors provide convincing evidence for an ordered distribution of Li, Cd and Ge atoms within tetra­hedral sites, which is not necessarily an easy task. The resulting structure is a lower symmetry, noncentrosymmetric derivative of the rarer family of diamond-like semiconductors based on hexa­gonal stacking. Although Pauling’s rules for ionic solids are recognized to have limitations (George *et al.*, 2020 ▸), they were applied to give a satisfying rationalization of the structural features, including support for the site distribution of cations. Crystallographers sometimes engage in what may appear to be esoteric debates (to outsiders) about whether a structure is centrosymmetric or not (Marsh, 1986 ▸). In the end, does it really matter? Within other contexts, such as chiral magnetism and superconductivity, this question is essential (Cheong & Xu, 2022 ▸; Smidman *et al.*, 2017 ▸); for optical materials, it is no exception. This leads to the third point. When microcrystalline samples of Li_4_CdGe_2_S_7_ are exposed to near IR light, a strong second harmonic generation (SHG) response is observed, giving a change in colour in the visible region, detectable by the naked eye. This observation clinches the assertion of a noncentro­symmetric structure. For practical use, NLO materials have to satisfy several other stringent conditions, including phase matchability, high wavelength transparency, high laser-induced damage threshold and ease of crystal growth. Li_4_CdGe_2_S_7_ shows promising characteristics, with an SHG response competitive with existing benchmark materials such as AgGaS_2_ but with a much improved laser-induced damage threshold.

Although many strategies have been proposed to ‘design’ materials having noncentrosymmetric structures, including com­putational and informatics approaches (Balachandran *et al.*, 2017 ▸; Song *et al.*, 2020 ▸), there is no substitute for experimental verification. Careful crystallographic analysis remains a useful guide for choosing the correct space group, but it is even better if the conclusions are supported by measurements of physical properties, such as the observation of NLO behaviour.
